# Sources of Error in Substance Use Prevalence Surveys

**DOI:** 10.1155/2014/923290

**Published:** 2014-11-05

**Authors:** Timothy P. Johnson

**Affiliations:** Survey Research Laboratory, University of Illinois at Chicago, 412 S. Peoria Street, Chicago, IL 60607, USA

## Abstract

Population-based estimates of substance use patterns have been regularly reported now for several decades. Concerns with the quality of the survey methodologies employed to produce those estimates date back almost as far. Those concerns have led to a considerable body of research specifically focused on understanding the nature and consequences of survey-based errors in substance use epidemiology. This paper reviews and summarizes that empirical research by organizing it within a total survey error model framework that considers multiple types of representation and measurement errors. Gaps in our knowledge of error sources in substance use surveys and areas needing future research are also identified.

## 1. Introduction

For the past 50 years, social and epidemiologic surveys have been employed to estimate and track the substance use patterns of representative samples of both adolescents and adults in the United States and other countries. Although many of these surveys are of exceptional quality and rigor (e.g., the Monitoring, the Future Survey, the National Survey of Drug Use and Health, and The Youth Risk Behavior Survey), for almost as long, there have been methodological criticisms and skepticism regarding their ability to accurately portray the behaviors they seek to measure [1–7]. Addressing these questions is important given the lack of alternative methodologies for efficiently monitoring substance use behavior within large national and subnational populations. The goal of this paper is to review and summarize the available empirical evidence addressing these questions, to identify gaps in our knowledge base regarding this issue, and to make some recommendations for needed future research to address those knowledge gaps.

## 2. The Total Survey Error Model

A useful framework for conceptualizing error in substance use surveys is the total survey error (TSE) model. The TSE model first delineated by Groves [8] focused on sampling, coverage, nonresponse, and measurement errors in surveys. This model successfully organized decades of empirical research within a single unifying theoretical framework. An expanded elaboration of the TSE model has been more recently presented by Lavrakas [9], in which he identifies two general classes of errors, measurement and representation, and then explores multiple subclasses of errors within each.  Table 1 lists the various elements of the Lavrakas TSE model.

Briefly, errors of representation are those concerned with technical problems that may impede a survey's ability to accurately mirror the population that the survey seeks to represent. These include failure to use sample frames that provide adequate coverage of the population being studied (coverage errors), imprecision in the sample(s) drawn from a sample frame (sampling error), errors associated with failure to contact or complete interviews with all sampled respondents, and failure to obtain answers to all questions included in a survey instrument (nonresponse errors), as well as failure to make adequate adjustments for complex sample designs and survey nonresponse (adjustment errors).

In contrast, errors of measurement involve failures to adequately assess the variables of interest in a survey. These include specification errors, which involve failures to correctly conceptualize survey constructs, and measurement errors, which include factors external to the construct being measured that nonetheless influence measurement quality. Processing errors are defects in the construction of survey data sets and/or final analytic variables and inferential errors which involve difficulties or failures when making adequate sense of the final survey data. The following two sections organize the empirical literature concerned with errors in substance use and misuse surveys within this TSE framework. Each of these error sources, of course, is broadly relevant to health survey research in general. Our goal here is to review their relevance specifically to substance use surveys.

## 3. Errors of Representation

### 3.1. Coverage Errors

Errors in coverage are generally a consequence of employing a survey sampling frame that does not include all individuals in the population being studied, or, alternatively, by employing methods that do not provide all members of the population of interest some probability of being sampled. As with all other elements of the TSE framework, this type of error is not unique to substance use surveys. Nonetheless, because likelihood of falling into a potential sample frame may in some cases be associated with substance use behaviors, substance use research may be uniquely vulnerable to coverage error.

In most community epidemiological surveys, there are many social groups that may be systematically excluded from commonly available sample frames. Some of these groups include homeless persons, individuals currently hospitalized, college students living in dormitories, persons incarcerated in the criminal justice system, and members of the military living on military bases. Substance use may be particularly high within some of these nonresidential populations [10, 11]. Weisner and colleagues [7] investigated this problem by comparing prevalence estimates from a general population community survey with data obtained from interviews with nonhousehold populations found in several inpatient and outpatient settings, such as alcohol, drug or mental health treatment, criminal justice, and/or welfare services. Not surprisingly, substance use was much more common among persons in these settings. For example, 11.3% of the household sample was defined as problem drinkers, compared to 43.1% of those found in nonhousehold agency settings. The disparities were even greater for indicators of weekly drug use (5.5% in the household sample versus 36.5% in the agency sample) and both problem drinking and weekly drug use combined (2.2% in household sample versus 18.7% in agency sample). Other research provides similar evidence of increased substance use and misuse among persons less likely to be sampled within single family households as part of community-based epidemiologic surveys [12].

There is also some evidence in the US that failure to incorporate cell-phone-only households into random digit dialed (RDD) telephone samples can lead to underrepresentation of young adults who are at higher risk for substance use behaviors. Delnevo et al. [13] found significantly decreased measures of binge drinking and heavy alcohol consumption between 2001–2003 and 2003–2005 in the national Behavioral Risk Factor Surveillance System (BRFSS) telephone surveys. Other research, employing the US National Health Interview Survey, which relies on face-to-face interviews, has demonstrated that adults in cell-phone-only households are more likely to report past year binge drinking behavior (37.6%), compared to those residing in households with landlines (18.0%), and to those in households with no telephone service (23.0%; Blumberg et al. [14]). The effects of excluding cell phone-only households from survey estimates of binge drinking are particularly serious for young adults (aged 18–29 years) and low income persons [15]. Similar relationships between type of phone subscribership and substance use reports have been identified in Australia [16] and in other US studies [17]. As rates of cell-phone-only residences continue to grow [18], the coverage error associated with excluding them from telephone samples will only increase, and it will become increasingly difficult to produce credible prevalence estimates using traditional landline-only sample frames.

School-based surveys are also subject to coverage errors, as substance use rates have been shown to be higher among adolescents who drop out of school [19, 20]. Hence, surveys of adolescents that are school based often underestimate substance use within this population, although it is important to acknowledge that many school-based surveys attempt to make no generalizations to nonschool populations. A recent analysis by Gfroerer and colleagues [21] using pooled data from the 2002–2008 NSDUH (National Survey of Drug Use and Health, previously known as the National Household Survey of Drug Abuse or NHSDA) surveys reported that substance use estimates were higher for most substances among school dropouts, compared to same-aged students. The effects of dropouts on overall estimates increased from the 8th to the 12th grades, as the numbers of dropouts increased. At the 12th grade level, they found that failure to account for dropouts would miss more than half of past year cocaine users, more than half of all lifetime Ecstasy users, 30% of current binge alcohol users, and 25% of current alcohol users.

Because school absenteeism is also known to be associated with increased substance use [22–25], Gfroerer and colleagues [21] additionally investigated the effects of school absenteeism on substance use prevalence estimates in the NSDUH. They reported that those students who missed more days of school were also more likely to be current alcohol users, binge drinkers, and marijuana users. In recognition of this problem, some surveys, such as the YRBS, conduct “make-up” sessions to maximize student opportunities to participate and minimize coverage errors.

### 3.2. Sampling Errors

Both probability and nonprobability sampling methods are commonly applied in substance use surveys. When probability sampling strategies are employed, all elements within the sample frame have a known, albeit not necessarily equal, probability of selection. The precision of survey statistics derived from such samples can be calculated with a good degree of confidence and used to estimate the sampling error associated with those statistics. All other things being equal, the size of a random survey sample is inversely associated with the degree of potential sampling error associated with it. The precision of survey estimates also decreases as probability samples deviate from simple random sampling designs, a commonplace occurrence designed to reduce survey costs. Of all the sources of total survey error, the sampling errors related to probability-based sample designs are probably the most well understood, and definable, in practice.

Nonprobability samples are commonly used when research questions focus on special populations believed to be at increased risk for substance use and misuse. There are a variety of well-known nonprobability, or convenience, sample designs commonly used in practice. One of the more popular approaches currently is known as respondent driven sampling (RDS), which was developed by Heckathorn [26, 27] and which has been used in numerous substance use studies [28–30]. Other popular nonprobability strategies in substance use research include venue and facility-based sampling [31–34], snowball sampling [35, 36], time-space sampling [37–39], and advertising for volunteers [40–42]. An important advantage of these designs is their cost effectiveness when researching rare or hidden populations, such as illicit drug users. Because probabilities of selection are unknown, however, there are no definable sampling errors associated with these designs. Rather, nonprobability based sample designs typically suffer from large coverage errors and sampling errors are completely unknown.

### 3.3. Nonresponse Errors

It is common knowledge that unit response rates in general population surveys have been declining for some time [43–45]. Survey response rates have been historically employed as a proxy indicator of survey quality in general and nonresponse error in particular [46]. Recent research, though, has demonstrated that response rates per se are not necessarily associated with nonresponse bias [47–49]. Rather, it is the degree to which survey respondents and nonrespondents differ from one another in terms of variables of interest to the survey, combined with the survey's response rate that defines nonresponse bias. A British study reported by Plant et al. [50], for example, compared two sets of survey data, with 25% and 79% response rates, respectively. No important differences in self-reports of alcohol consumption were found between the two.

When considering substance use behaviors, there are reasons to be concerned about differences between survey respondents and nonrespondents. Pernanen [4] many years ago suggested that persons who drank heavily might be more difficult to contact as part of survey efforts and would be less likely to cooperate when contacted. In a Canadian survey, De Lint [11] reported that more in-person contact attempts were required to interview those respondents who reported greater numbers of purchases of alcoholic beverages. Cottler et al. [51] additionally reported that those respondents diagnosed with alcohol abuse and dependence required greater numbers of contact attempts in order to complete interviews. Crawford [10] also reported more alcohol consumption among those respondents most difficult to contact. Using a population register in Sweden, Tibblin [52] found higher rates of survey nonparticipation among middle aged men who were known to have experienced alcohol related problems. There is also some general evidence that survey nonresponse is greater among persons with poor health [53, 54]. A Swedish study has reported that survey respondents were less likely to have been hospitalized with alcohol diagnoses, compared to nonrespondents [55]. These findings are generally interpreted as evidence that heavy drinking may be a barrier to participation in social surveys due to difficulty in making contact and also in convincing those individuals who are contacted to agree to participate [56]. Other investigations, though, have reported no differences in alcohol use between those who do and do not participate in epidemiologic surveys [57–60], and alcohol abstainers have also been found to be underrepresented [56].

It should also be noted that standard field procedures in many surveys actually exclude active substance users from participation. Much research explicitly requires interviewers* not* to conduct interviews with individuals who are visibly intoxicated or appear high on other substances. Kish [61] commented on this problem nearly 50 years ago, referencing a case in which a respondent was drunk by the time they came home after work every day throughout a survey's field period. While such protocols are necessary for orderly data collection and are invoked only infrequently in practice, the potential effects of such protocols on nonresponse bias must nonetheless be considered. In addition, despite some claims to the contrary [62], knowledge that a survey is concerned with substance use appears to have no effect on respondent willingness to participate [55, 63].

Other relevant information comes from studies of attrition in panel surveys, in which the same respondents are interviewed at multiple time points. A number of such investigations have documented higher levels of attrition among high alcohol and drug users [64–73]. In contrast, some other research has found higher attrition among nonusers [74], and Thygesen and colleagues [75] have found both high alcohol intake and abstinence to be associated with increased likelihood of panel attrition. In their study, attrition was also found to be predictive of increased mortality from alcoholic liver cirrhosis and alcoholic liver diseases. Still other research has found no differences between those who do and do not drop out of panel studies [76].

Evidence from research specifically designed to assess nonresponse bias is also informative. Several types of nonresponse bias studies are routinely conducted. One type is known as follow-up surveys, which typically involve attempting to obtain survey data from nonrespondents to the primary survey [77]. Caspar [57], for example, conducted follow-up face-to-face interviews with a sample of nonrespondents to the 1990 NHSDA, concluding that initial nonrespondents were more likely to report lifetime drug use. Lahaut et al. [56] provide an example of a nonresponse follow-up survey with individuals who initially did not respond to a mail survey and who were subsequently visited by interviewers to complete a face-to-face interview. These analyses suggested that abstainers were underrepresented in the initial survey. Hill et al. [78] report a telephone follow-up survey of nonrespondents to a primary mail survey. They also found lower reporting of unsafe alcohol consumption among initial nonresponders. Lemmens et al. [60] conducted a telephone follow-up survey of nonrespondents to a face-to-face survey, concluding that there were only small effects of nonresponse on self-reporting of alcohol consumption. An important potential limitation when interpreting findings from follow-up surveys such as these is the use of different modes of data collection between the primary survey and the follow-up effort. Given what is known about mode differences in reporting of substance use behaviors (see Section 4.2), it would not be surprising that a telephone follow-up to a self-administered survey might suggest that the initial survey overestimated substance use, whereas a self-administered nonresponse follow-up survey to an initial interviewer-assisted effort might suggest that it had underestimated substance use. In each case, the effects being attributed to nonresponse bias may actually be a consequence of mode differences rather than systematic nonresponse. Indeed, there are several examples in the literature of surveys that relied on interviewer-assisted follow-up interviews (cf., Hill et al. [78]; Lahaut et al. [56]) that produced data suggesting that primary survey respondents overreport substance use behaviors.

Examples of other types of nonresponse bias analyses that focus on respondent substance use patterns include studies that compare early versus late respondents [56, 79–81]. An example is a study reported by Zhao et al. [62], who compared the answers of persons responding early and late to the Canadian Addictions Survey. Respondents were more likely to have higher incomes and to be educated, males, young adults, and substance users. Such studies employ a continuum of resistance framework that assumes that respondents who require greater effort to contact and interview are more similar to nonrespondents than are those who initially agree to survey requests [82]. Other strategies compare estimates from multiple surveys [62], compare frame data for respondents, nonrespondents, and the full sample [60], or compare estimates from surveys that have high versus low response rates [50].

Another useful strategy for assessing nonresponse bias is to supplement survey data with information obtained from other sources, such as administrative records. For example, Gfroerer et al. [83] examined response patterns in the 1990 National Household Survey on Drug Abuse by merging survey findings with records from the 1990 Decennial Census. Of course, this required special authorization from the government, given the strict data protections associated with the census. They found that persons with some characteristics known to be associated with substance use (i.e., living in urban areas, being male) had lower response rates and that persons with other characteristics believed to be associated with nonsubstance use (older age and higher income levels) also had lower response rates and concluded that these various nonresponse correlates would likely cancel out much of the bias either set might have introduced into the survey estimates.

Finally, it is also important to recognize that high nonresponse rates to individual survey questions (a.k.a., item nonresponse) may also be an indicator of data quality problems in substance use surveys. Some research suggests demographic variability in nonresponse rates to substance use questions. Owens et al. [84] found that African Americans and persons who were separated or divorced were less likely, and females and persons aged 55 and older were more likely, to answer questions concerned with their use illicit drugs. Increased item nonresponse rates to substance use questions among minority groups have also been reported by Witt et al. [85], although Aquilino [86] reported no differences. An item nonresponse study of adolescents additionally found higher nonresponse rates to questions concerned with alcohol and marijuana use among male, compared to female respondents [87].

### 3.4. Adjustment Errors

Errors of adjustment involve failures to account for the potential effects that a survey's sample design and execution may have upon empirical findings. These may include instances in which sample weights fail to incorporate all sample design and/or nonresponse factors, when variances are unadjusted for the clustering of respondents within sampled geographic areas, or when the available sample weights are not correctly used. An unfortunate example of the failure to properly employ sample weights occurred about a decade ago when a report concerned with illegal sales of alcohol to underage minors in the US seriously overestimated the proportion of all alcohol sales that were reportedly being made to underage youth. The researchers were conducting a secondary analysis of a public release version of the 1998 NHSDA and failed to weigh their data for the survey's stratified sample design, in which young persons aged 12–20 were significantly oversampled. Because only persons under the age of 21 purchase alcohol illegally in the US, their overrepresentation in the unweighted NHSDA data file led to an overrepresentation of illegal sales in those data. This was an error that could have easily been avoided through the use of the preexisting sample weights. The erroneous findings, which were reported nationally, were quickly exposed as flawed [88].

Failure to employ nonresponse weights when survey response rates across different demographic subgroups vary considerably and those same variables which are correlated with substance use patterns can also result in biased substance use estimates. In addition, adjustment errors associated with clustered sample designs (when clustering is not taken into account) can lead to survey estimates with artificially small standard errors that can be misinterpreted as being overly precise [89]. In general, avoidance of adjustment errors would seem to require analysts who possess both substantive knowledge of the addiction processes being examined and methodological knowledge and expertise regarding complex sample design and analysis procedures.

## 4. Errors of Measurement

### 4.1. Specification Errors

When survey questionnaires do not correctly conceptualize and/or operationalize constructs of interest, they are understood to have specification errors. These can take several forms. For example, the street terminology used by drug users is often unique, constantly changing, and varies across locations. Not surprisingly, research demonstrates that the drug names employed in survey questionnaires are not always consistent with the names employed by users in the community [90, 91]. The continued introduction of new substances of course also contributes to specification errors.

In order to adequately assess substance use, it is necessary to ask respondents about all the forms of alcohol and/or drug they may have consumed. Hence, survey questions intended to measure any alcohol or drug use must be able to capture experience with each form of these substances. Global questions that ask about use of the substances in general can be expected to miss some experiences with less common varieties of each. Although these points may seem obvious, they can lead to specification errors more often than most researchers would prefer to admit. Avoiding specification errors requires careful attention during the instrument design process to the specific goals for which the survey is intended to be used.

### 4.2. Measurement Errors

Measurement error occurs when survey questions fail to measure what they were designed to measure. There are several potential sources of measurement error which must be considered when constructing a survey instrument or analyzing survey data. Broadly speaking, these include design effects, respondent effects, interviewer effects, and context effects.

#### 4.2.1. Design Effects

Virtually every element of a survey that is exposed to respondents is likely to provide them with cues regarding the information being sought [92]. Although many if not most of these cues are unintentional from the researcher's perspective, they can nonetheless be expected to influence self-reports in ways that cannot always be anticipated or controlled. We refer to these as design related errors. Some important design issues discussed below include methods for asking about substance use, mode effects, use of skip patterns, and reference periods. Other design factors that may influence measurement quality include how clearly a survey is introduced as being concerned with substance use, the survey's sponsor, the procedures employed to obtain respondent informed consent, the use of incentives, and the survey's focus as either primarily concerned with substance use or concerned with a more broad set of topics [21]. Regarding this later point, it has been suggested that survey respondents are more willing to discuss negative personal behaviors when they are also asked to report about positive personal behaviors and characteristics [93]. 

*Methods for Asking about Substance Use*. Of course, the wording and structure of survey questions can be expected to have a strong influence on the answers obtained, and experimental comparisons have revealed differences in the magnitude of substance use reports obtained using various question measurement strategies. Kroutil et al. [94], for example, have documented the fact that open-ended questions seriously underestimate drug use prevalence rates. Other research has compared methods for measuring alcohol consumption. Rehm et al. [95] has reported findings from a within-subjects experiment that documents consistently higher prevalence rates for several indicators of harmful drinking when graduated-frequency measures [96] are used, in comparison to the more commonly employed quantity-frequency question response format [97, 98], and weekly drinking recall questions [95]. Other studies have also found graduated-frequency measures to produce higher estimates of alcohol use in comparison to quantity-frequency measures [99, 100]. The superior performance of the graduated-frequency format appears to be based on its ability to more precisely measure irregularly high levels of consumption, although there is some evidence suggesting that the graduated-frequency approach may actually overestimate consumption [100, 101]. Other less commonly used measurement strategies, such as the* yesterday* (or* recent recall*) method of reporting in which respondents are asked to report on their alcohol use during the previous day only, have been found to produce higher estimates than either the quantity-frequency or graduated-frequency measures [102]. The use of a daily diary protocol for collection of alcohol consumption is frequently considered to be a “gold standard” measurement approach [100, 103], but not very practical for most survey applications.

The design of response categories for use in quantity and frequency questions can also influence respondent self-reports. For example, Schwarz [104] has shown how simple changes in the sets of response options presented to respondents, such as emphasizing low versus high frequency events or behaviors, can have effects on overall response patterns. Indeed, Poikolainen and Kärkkäinen [105] have reported obtaining higher alcohol consumption reports when employing quantity and frequency questions that include more heavier intake response options.

It is somewhat ironic that quantity-frequency measures remain commonly utilized in practice, despite the fact that it is conventional wisdom among most substance use researchers that alcohol and drug consumption behaviors are far more variable across even brief time intervals than are assumed by these questions [92, 106]. By their very nature, quantity-frequency items ask for average amounts of use, essentially insuring that they will not capture episodes of heavy or binge drinking. Hasin and Carpenter [107] have documented in a community sample that as many as 30 percent of all respondents report having difficulty when answering typical survey questions concerned with usual drinking patterns due to changes in their drinking behavior during the time period in question and that this problem was particularly acute for persons with symptoms of alcohol dependence. The key advantages of the quantity-frequency measures that make them continue to be popular are their simplicity, ease of answering, and the relatively small amount of space they require in survey instruments. L. C. Sobell and M. B. Sobell [98] and Bloomfield et al. [101] provide comprehensive overviews of the strengths and limitations of various approaches to measuring alcohol consumption in survey questionnaires.

*Substance Use Reference Periods*. Various reference periods are used to restrict and specify the time intervals for which respondents are asked to retrospectively report their substance use activities. Most often used in practice are 30-day and 12-month reference periods, although there are many variations. Each has its own advantages and disadvantages. It is common knowledge that recall accuracy decays with increasing length of these time intervals [108], as research suggests that greater alcohol prevalence is obtained when shorter reference periods are employed in survey questions [109, 110]. Although more susceptible to recall concerns, a 12-month recall period would have the advantage of being less affected by seasonal variations in substance use [92, 111]. A 30-day reference period, in contrast, might be less likely to capture binge drinking episodes. Hence, some surveys may ask questions about multiple reference periods in order to address the limitations of each.

Also problematic are questions concerned with age of initiation of alcohol and other drug use. Age of first substance use, of course, is considered an important risk factor for subsequent substance abuse, and accurate measurement is hence important [112]. Unfortunately, the length of recall necessary to correctly answer this question can be problematic for many respondents. Forward telescoping, in particular, when respondents underestimate the length of time since an event took place, is an important threat to the quality of self-reports of age of first use [113]. Numerous studies have documented problems with accurate recall of this information [64, 114–121].

*Questionnaire Skip Patterns*. A common issue when designing substance use questionnaires is the question of whether it is best to employ skip patterns, which allow respondents to avoid answering follow-up questions that are clearly not applicable to them or to instead require all respondents to provide all answers to all items. The rationale for requiring responses to all items is twofold. First, there may be privacy concerns associated with the use of skip patterns, as those who report substance use will require more time to complete all follow-up questions, presumably allowing interviewers and/or other observers to conclude that they are in fact substance users. Second, although it is somewhat burdensome for respondents, it is likely that the presence of skip patterns will be quickly detected by many respondents and possibly motivate some to provide negative answers to filter questions in order to “skip out” longer blocks of questions that request details regarding substance use experiences. As an example of a skip pattern, a question that asks respondents if they have ever used marijuana might be employed as a filter item. Those respondents indicating that they had used marijuana would then be eligible to answer a series of follow-up questions that queried about frequency of use, age of initiation, and so forth. In contrast, avoidance of skip patterns would require respondents to answer all follow-up questions, typically by selecting a “have never used marijuana” response option, which would be available for use with each follow-up question. Such an approach can considerably increase the burden and amount of time necessary to complete a questionnaire for nonusers of the substances being examined. The NSDUH has historically not employed skip patterns. An experiment reported by Gfroerer et al. [83] investigated the effects of using skip patterns as part of the NHSDA. In their random experiment, they found significantly lower prevalence rates for the five illicit drugs examined when skip patterns were employed. Because no differences were found in alcohol use estimates, it was concluded that privacy concerns associated with answering the most sensitive questions was a more likely explanation for the findings.

*Mode Effects*. Survey data can be collected using a variety of modalities, including self-administered paper-and-pencil or electronic questionnaires, and telephone or in-person interviews. The presence of mode effects in surveys is well recognized, and there is now a considerable body of evidence documenting the effects of mode on the quality of self-reports of substance use behaviors. In general, survey modes that rely on respondent self-administration are found to obtain greater reports of alcohol and drug use than do those modes that require interviewers to directly ask about use of these substances [55, 58, 122–130]. There is additionally some evidence that these mode effects are greater for more sensitive illicit substances, such as cocaine and marijuana, compared to alcohol use [131].

Among self-administered modes, audio-computer-assisted-self-interviews (ACASI) appear to generate higher reporting of substance use behaviors than do paper-and-pencil (PAPI) self-administered answer sheets [132, 133]. Computer-assisted questionnaires produce data that is more internally consistent and more complete, helping to reduce the need for editing, imputation, and other processing activities that may lead to processing errors (see Section 4.3) [133]. Research has also begun to explore the reliability and validity of substance use surveys conducted via the internet. Eaton and colleagues [134] randomly assigned classes of high school students to respond to PAPI or web questionnaires, concluding that there were few differences in prevalence estimates obtained across the two modes. Ramo and colleagues [135] examined the quality of self-reported marijuana use in a convenience sample of young adults who completed a web-based questionnaire, concluding that such data can be reliably collected. Bauermeister et al. [28] have reported on the use of respondent driven sampling to more systematically sample young adults to participate in a substance use survey. Other investigators have compared internet reporting of alcohol use with reports obtained from self-administered mail questionnaires and both face-to-face and telephone interviews, concluding that online reports have similar levels of measurement quality [136–138].

Among interviewer assisted modes, some evidence suggests that face-to-face interviews appear to produce greater reports than do telephone interviews [86, 123, 139, 140], other evidence suggests no differences in substance use estimates between these two interviewer assisted modes [141, 142], and one study suggests that higher rates of some alcohol-related measures can be obtained by telephone [143]. Some research has also investigated the use of interactive voice recording (IVR) systems (a.k.a., “T-ACASI”—telephone audio computer-assisted self-interviewing) to improve the quality of substance use data collected by phone [144, 145].

#### 4.2.2. Respondent Effects

Survey respondents vary considerably in their abilities and willingness to provide accurate answers to questions regarding substance use behaviors. Respondent behaviors can be understood within the framework of the generally accepted cognitive model of survey response [146], which recognizes four basic tasks required from respondents when they are answering each survey question. These include (a) question interpretation, (b) memory retrieval, (c) judgment formation, and (d) response editing. This is a useful model for understanding how variability across respondents may influence the quality of self-reported substance use information. Evidence regarding how three of these information processing tasks may influence the quality of substance use behavior reporting is reviewed below.

*Question Interpretation*. Because respondents sometimes employ substance use terminology that differs from that employed in research questionnaires [91, 147], the risk of miscommunication may be greater in substance use surveys, compared to other topics. The complexity of some substance use terminology may also sometimes lead to respondent confusion. This may be of particular concern in surveys of adolescents, who may not always have sufficient knowledge to correctly respond to questions regarding the use of various drugs [147–149]. Johnston and O'Malley [150] have presented evidence suggesting that respondents sometimes are more likely to deny, or recant, ever having used certain substances that they had previously reported having used (see also additional discussion of recanting in section below on Response Editing). Of particular relevance here is their finding that recanting varies by type of drug being asked about, with the recanting of tranquilizers and barbiturates found to be greater than that for marijuana and cocaine, a finding that they suggest to be related to the complexity of the definitions of these two substances, relative to marijuana and cocaine definitions, which of course also have some complexity. In alcohol research, recent reviews have found that respondents commonly misinterpret standard drink sizes, suggesting that alcohol intake may be systematically underestimated in survey research [151, 152].

A related concern is the degree to which respondent cultural background may influence the interpretation and/or comprehension of survey questions. Substance use patterns and practices are known to vary cross-culturally [153–155], and those varied experiences and beliefs regarding substance use can also be expected to influence respondent knowledge and familiarity with the topic in general and related terminology in particular. Experienced researchers, of course, recognize the importance of investigating and addressing these potential problems by employing focus groups, cognitive interviews, and ethnographic methods during survey development (c.f., Gardner and Tang [156]; Midanik and Hines [157]; Ridolfo [158]; and Thrasher et al. [159]).

*Memory Retrieval*. The accuracy of respondent recall has been the focus of much attention among methodologists [160, 161] and has been historically considered one of the more common explanations for inaccurate reporting of substance use behaviors [4, 120, 121]. Indeed, when answering survey questions concerned with substance use, the retrieval of the memories necessary to report accurately can be particularly difficult for several reasons. Poorly worded survey questions may present respondents with difficult cognitive challenges in terms of the effort necessary to retrospectively retrieve specific and/or detailed information that may not be readily accessible in memory [81]. There is also evidence that heavy drinking [4, 162], cocaine [163, 164], and MDMA use [165–167] may be associated with impaired memory. Mensch and Kandel [168] have found inconsistent reporting of marijuana use to be associated with degree of drug use frequency, with the more involved users providing less consistent survey responses, a finding they associate with faulty memory. Although considerable research has been invested in experimenting with strategies for aiding respondents with memory retrieval in general [169, 170], few efforts have focused on aiding recall of substance use information. Hubbard [171], however, has reported a series of experiments that used anchoring manipulations to improve respondent recall, although these were not found to be very effective.

*Response Editing*. Once respondents have successfully interpreted a survey question and retrieved the relevant information necessary to form an answer, they must decide whether that answer is to be accurately shared with the researcher. Given the illicit and sometimes stigmatizing nature of substance use behaviors, conventional wisdom often suggests that some respondents will make conscious decisions to underreport, or deny altogether, any such behavior [4]. That survey respondents will sometimes attempt to present themselves in a favorable, albeit not completely accurate, light during survey interviews is well understood and is commonly referred to as social desirability bias. Concerns about the potential effects of social desirability bias have been the subject of considerable research in the survey methodology literature [172–175]. In general, respondents are known to overreport socially desirable behaviors, such as voting [176] and exercise [177], and underreport socially undesirable behaviors, including drug and alcohol use [178]. Bradburn and Sudman [172] have explored and documented the sensitive nature of substance use questions by asking a national sample of respondents in the US how uneasy discussing various potentially sensitive topics would make them feel. They found that 42.0 percent reported that they believed most respondents would be “very uneasy” discussing their use of marijuana and that 31.3 and 29.0 percent, respectively, would also be uneasy discussing stimulant and depressant use, and intoxication. Only 10.3 percent indicated that they believed most people would be uneasy discussing drinking in general. This survey, though, was conducted more than 30 years ago and it is unclear to what degree these topics would elicit similar feelings of discomfort today.

Respondents may be uneasy discussing their substance use for several reasons, including the need to avoid the social threat and feelings of shame and embarrassment associated with violating social norms [179, 180]. Reporting illicit substance use may also be viewed by some respondents as a sign of weakness and, hence, something not to disclose [181]. These points are consistent with research findings that indicate that substance use underreporting increases with the perceived stigma of the substance being discussed [182–184]. Respondents may also elect not to admit to substance use behaviors in order to avoid potential legal sanctions, out of fear that a breach of confidentiality might risk their employment or reputation, and/or because they believe that such information is highly personal and not to be shared. Some research suggests that questions about current use of illicit substances are more likely to produce underestimates when confidentiality is less certain, compared to questions concerned with past use [185]. Experimental studies that have compared substance use reporting patterns when provided with assurance of anonymity versus confidentiality have generally found few differences across conditions [186–188].

Some measures of the propensity to provide socially desirable answers have been found to be associated with substance use reporting such that likelihood of providing socially desirable responses in general is associated with less likelihood of reporting alcohol and/or drug use behavior [172, 189–191]. These findings have been interpreted alternatively as (a) evidence that underreporting of substance use is a consequence of respondent attempts to conceal illicit behavior or as (b) evidence that persons who engage in socially desirable behaviors in general also report, accurately, that they do not engage in substance use behaviors. Although this question remains unresolved, we note that other research has demonstrated the absence of an association between one measure of social desirability, the Crowne-Marlowe scale [173], and a measure of cocaine use underreporting that was based on comparisons of self-reports with biological assays [192].

The accuracy of self-reports of substance use behaviors may also vary by the race/ethnicity of the respondent. A literature review of 36 published studies conducted in the US found consistent evidence of lower reliability and validity rates of substance use reporting among racial and ethnic minority populations [193]. More recent studies have reported similar findings [194, 195]. The specific source of these differences, however, is not clearly understood. Models that have been proposed suggest that greater reporting errors among minority groups may be a consequence of differential group educational achievement and question comprehension, greater minority concerns with privacy, discrimination and risk of prosecution, and/or stronger effects of social desirability pressures on minority groups to report behaviors that conform to majority cultural values. Internationally, cultural differences in normative patterns of alcohol consumption and other substance use may also influence degree of response editing. In nations where wine is considered part of a meal, rather than mood-altering substance, underreporting might be expected to be much less of a concern.

One limitation in much of the research reviewed here is the assumption that greater self-reports of substance use behaviors are more valid [196, 197]. Indeed, overreporting is another measurement concern [197, 198]. There have been cases of respondents providing daily alcohol use reports that are physically impossible [4]. In surveys of adolescents, there is also a widespread belief that some respondents overreport their alcohol and other drug use, possibly to impress peers and improve one's social status or as part of a general desire for attention [3, 149, 199–202]. Gfroerer and colleagues [21] have speculated that such overreporting of substance use might be more likely to happen during school-based surveys, usually conducted in classroom settings, where peers may be more likely to be aware of respondent answers. It has also been suggested that respondents may in some situations elect to present themselves in a highly negative manner, perhaps for personal amusement or to obtain treatment services [11, 148, 203, 204]. In an effort to identify such overreporters, several investigators have asked respondents about their use of substances that do not exist [205]. It is notable that these studies have found very low self-reported rates of use of these fictitious substances. Petzel et al. [206], for example, found that 4% of his sample of high school students reported the use of the nonexisting drug “bindro.” They also found that those who reported the use of a nonexistent drug also reported more use of all other drugs included in their survey, compared to those who indicated, correctly, that they did not use “bindro.” Others have reported similar findings when asking survey respondents about the use of nonexistent substances [202, 207–209]. Of course, it may be that heavy drug users just assume, incorrectly, that they have used all available substances at one time or another in their past.

Others have questioned whether or not it is correct to assume that all substance users will be hesitant to accurately report on their patterns of use. Wish et al. [210], for example, have suggested that heavy substance users may be less concerned about social and other consequences of reporting such information. Interviews with persons receiving treatment, though, have found little interest in publicly discussing their patterns of use [211].

Concern with the accuracy of substance use reporting has led to a variety of attempts to validate or corroborate survey responses. For example, several panel surveys have demonstrated considerable stability in respondent reporting of substance use over time [22, 212, 213]. Research, however, has also investigated the recanting of drug and alcohol use, which is the tendency of some panel survey respondents to claim no lifetime experience with a given substance, when they have previously reported having used it [200]. Recanting has been identified in responses to both alcohol [214] and drug use questions [119–121, 150, 201, 215–220]. Depending on the age group being surveyed (adults versus adolescents), recanting may represent deliberate efforts to deny previously reported activity, exaggerations regarding behaviors that never actually took place, poor comprehension of survey questions during at least one wave of interviews, poor recall of information, or simple carelessness when answering [200, 217]. Research by Martino et al. [221] suggests that recanting is a consequence of both deliberate misreporting and errors in understanding of survey questions. In surveys of adolescents, one possible explanation for recanting is that younger and less mature respondents may be more likely to exaggerate substance use during surveys conducted in classroom settings in which peers might be aware of one another's answers and that they may then provide more accurate answers during subsequent survey waves as they subsequently become more mature [215]. Interestingly, longitudinal follow-ups with Monitoring and the Future Survey respondents have found that recanting is greater among adults with occupations that might be expected to strongly sanction the use of illicit substances, such as those associated with the military and law enforcement [150]. Percy et al. [201] have also documented increased recanting among adolescents who had received drug education during the study period, suggesting a potentially biasing effect of education on self-reports. Higher recanting among low level substance users has also been reported [201, 216].

Other research has sought to validate self-reported substance use behavior by comparing those reports to toxicological findings from biospecimens collected at the time that interviews are conducted. One of the first studies conducted with a community sample (in Chicago) by Fendrich et al. [222] indicated that recent cocaine and heroin use estimates obtained from hair testing were considerably higher than were self-reports obtained from the same respondents. A follow-up survey found that higher rates of cocaine and heroin were obtained from drug assays of hair, saliva, and urine samples, compared to self-reports from respondents to a community survey [178]. A higher estimate of marijuana use, though, was derived from self-reports, compared to drug test assays, a finding that was interpreted as evidence of the limitations of hair testing for the detection of marijuana use. Similar findings of underreporting of cocaine and heroin have also been obtained from general population surveys conducted in Puerto Rico by Colón and colleagues [223, 224] and of men who have sex with men in Chicago [225]. Another study conducted as part of the NSDUH investigated agreement between self-reported use of marijuana and cocaine and urine tests concluded that “most youths aged 12 to 17 and young adults aged 18 to 25 reported their recent drug use accurately” ([226] page 4). Ledgerwood et al. [195] examined the association between hair testing and self-reported illicit drug use, concluding that agreement between tests and self-reports to be substantial for marijuana and cocaine, moderate for opiates, and fair for methamphetamines. Other research has employed urinalysis [227] and hair assays [228] to document drug use frequency underreporting among drug users receiving treatment. While providing valuable insights, it is important to acknowledge that each of these sources of confirmatory biological information is also imperfect measure of substance use, suffering from a variety of limitations, including imprecise and variable detection windows, vulnerability to contamination, and individual and race/ethnic group variability in rates of chemical absorption and retention [229, 230].

Another approach to validating self-reports of substance use is to compare information obtained from respondents with those of significant others, a strategy that has found good but far from perfect levels of corroboration [202, 208, 231–233]. Parents and children have also been asked to corroborate one another's reports of alcohol use. In a Dutch study, Engels et al. [234] found that both children and parents underestimate one another's alcohol consumption to some extent and that underestimation of adolescent alcohol consumption by parents was related to lack of knowledge and control of their children's activities. An important caveat when employing this approach is that proxy and self-reports generally suffer from the same sources of error [235]. Interestingly, perceptions of untrustworthiness by others have also been found to be associated with drug use recanting among adolescents in a study reported by Weinfurt and Bush [236].

An aggregate level strategy for evaluating self-reports of alcohol use is through comparisons of alcohol sales and tax information. A number of studies have taken this approach and have consistently found evidence suggestive that survey self-reports in some cases vastly underestimate total alcohol consumption [237–240]. State-level estimates from self-reports, though, do correlate fairly strongly with the estimates from sales/tax data, suggesting sensitivity to variations in substance use behavior [238]. One recent study that compared self-reports of alcohol purchases, rather than self-reported alcohol consumption, found much closer agreement between total estimates developed from those self-reports in comparison to total retail alcohol sales in Sweden [241]. Interestingly, this study also found considerable variability by type of alcohol, with sales of wine far more accurately reported than beer and spirits, suggesting the possibility that social desirability concerns may be at least partially responsible, given that wine is likely viewed as a more socially desirable alcoholic beverage, at least in the Swedish context. Reporting of wine consumption was also found to be more complete in a Canadian study [242].

One strategy designed to provide respondents with greater privacy when speaking with interviewers about highly sensitive questions such as substance use behavior is the randomized response technique, first proposed by Warner [243]. Several studies have documented the usefulness of this procedure among both students and adults. Goodstadt and Grusin [244] found higher drug use reporting for five of six substances among high school students in Ontario. Weissman et al. [245] compared substance use self-reports obtained with and without the use of the randomized response technique during telephone interviews conducted as part of a general household survey in New York City and also found increased reporting for three of four substances when using the randomized response technique. An important drawback noted, though, was that only 52% of those randomly assigned to respond using this technique actually agreed to do so. In contrast, McAuliffe et al. [246] reported no differences in reports of illicit drug use among those responding via the randomized response technique, compared to those answering direct questions. Some limitations of this technique include the challenge of correctly administering it in practice and its ability to provide aggregate estimates only [199].

The bogus pipeline is another approach that has been employed in attempts to induce more accurate reporting of substance use behavior. This involves the ethically questionable practice of leading respondents to believe that their questionnaire responses will be validated using some alternative means, when in fact the investigator has no intention of doing so. Rather, the implied threat of validating respondent answers is used to exert pressure on respondents to answer more truthfully. In general, however, the use of the bogus pipeline procedure has failed to obtain higher estimates of substance use behavior, at least among adolescents [247–249]. A meta-analysis has confirmed the nonefficacy of the bogus pipeline procedure for improved reporting of alcohol consumption and marijuana use [250]. One subsequent study, by Tourangeau et al. [251], did however demonstrate the effectiveness of the bogus pipeline technique for increasing respondent reporting of sensitive behaviors, including alcohol and illicit drug use. In addition, a special population study has suggested that the bogus pipeline procedure may be successful in improving self-reports under certain conditions. Lowe et al. [252] found that, among pregnant women, those randomly assigned to a bogus pipeline condition were nearly twice as likely to report alcohol consumption when completing a self-administered questionnaire.

Finally, when considering respondent related reporting errors, it is highly likely that multiple sources of respondent related reporting errors are operating simultaneously. For example, Johnson and Fendrich [253] demonstrated, using latent measures of cognitive processing difficulties constructed using debriefing probes, that social desirability concerns were predictive of discordant drug use reporting and drug use underreporting, while memory difficulties were predictive of drug use overreporting.

#### 4.2.3. Interviewer Effects

Interviewers can introduce errors by misreading questions, failing to probe answers correctly, not following other elements of standardized survey protocols, and by deliberate falsification of survey interviews [254, 255]. Interviewer affiliation with governmental agencies may also influence respondent willingness to report substance use behaviors [256]. Interestingly and somewhat counterintuitively, interviewers with no prior project-related experience have been found to generate higher levels of marijuana and cocaine reporting in a national substance use survey [130, 257]. Research by Chromy et al. [258] also finds that more experienced interviewers achieve higher response rates, in addition to eliciting fewer reports of substance use behaviors, suggesting they may be more successful in gaining cooperation from nonsubstance users who might find a survey on this topic to be less personally salient or interesting, although they do not believe that this fully accounts for the observed differences, which remain unaccounted for.

Another possible mechanism that may account for interviewer effects involves social distance. It is possible that the social distance between respondents and interviewers may influence respondent willingness to report sensitive behaviors such as substance use. Johnson and colleagues [259] found that adult respondents in a telephone survey regarding substance use treatment needs in Illinois were more likely to report recent and lifetime drug use when respondent-interviewer dyads were characterized as having relatively little social distance. In that study, social distance was measured using a simple count of the number of shared demographic identities (i.e., same gender, same race/ethnicity, similar age, and similar educational attainment). Johnson and colleagues [260] also explored the effects of social distance between race/ethnic groups in a study in which they probed respondents regarding how comfortable or uncomfortable they would feel when interviewed about their alcohol consumption patterns by interviewers from the same and from other cultural groups. When asked how they would feel if interviewed by an interviewer with the same background, large majorities of African American (88.8%), Mexican American (74.7%), Puerto Rican (85.9%), and non-Hispanic white (92.9%) respondents indicated they would feel comfortable. However, when asked how they would feel if the interviewer asking about their alcohol use was from another cultural group, the proportions indicating they would continue to feel comfortable decreased to 60.0% of African Americans and Mexican Americans and 69.4% of Puerto Ricans. Among non-Hispanic whites, though, the proportion indicating they would continue to be comfortable remained very high (89.3%), suggesting group differences in reactions to interviewers of similar versus different race/ethnic backgrounds.

Other research has also examined the effects on substance use reporting of similarities and differences in various demographic characteristics between interviewers and respondents. In studies conducted in Iowa many years ago, female respondents were more likely to report alcohol consumption to male interviewers, and conversely, male respondents were more likely to report alcohol use to female interviewers [261]. Johnson and Parsons [262] found that homeless respondents were more likely to report drug use to male interviewers, a finding that they linked to a “likely user” hypothesis that suggests that male interviewers were more likely to elicit positive substance use reports because their gender is perceived as being more likely to be substance users themselves and more tolerant of substance use by others. In contrast, a study conducted by Darrow and colleagues [263] reported that gay males were more likely to report drug use to female interviewers, who were viewed as having greater empathy and sympathy for deviant behavior than would male interviewers. In a survey conducted in The Netherlands, higher rates of alcohol use were reported by Turkish and Moroccan respondents to Dutch interviewers, compared to interviewers who were ethnically matched [124]. These researchers also hypothesized that minority respondents may have either (a) exaggerated their alcohol consumption to comply with the perceived norms of the person interviewing them or (b) underreported, or denied altogether, the use of alcohol when interviewed by interviewers from an Islamic background who would have been perceived as having a far less permissive opinion of alcohol use. This limited evidence does not suggest a clear pattern of effects of any interviewer characteristics on respondent self-reports of substance use behaviors, although it does seem likely that interviewer characteristics do matter in many situations.

Interviewer-respondent familiarity with one another may also influence the quality of self-reported substance use behaviors. For example, Mensch and Kandel [168] found that, in the panel survey of National Longitudinal Survey of Youth, marijuana use reporting was lower among respondents who had been interviewed more times previously by the same interviewer, suggesting that interviewer familiarity cued respondents regarding social desirability expectations, which depressed their drug use reporting. Ironically, again, the use of experienced survey interviewers, something that would typically be considered an important strength of any study, would appear in some circumstances to be a factor contributing to lower quality data, at least when interviewers are serially assigned to the same subsets of respondents.

#### 4.2.4. Context Effects

Various aspects of the social and physical environment within which survey data are collected may also influence the quality of the information collected. One aspect of the social environment that has received attention is the absence or presence of other individuals during the interview, as this is believed to influence the social desirability demands or pressures that respondents may perceive. In general, the presence of others during survey interviews is known to be associated with lower reporting of sensitive behaviors, including substance use. In an early study, Wilson [81] noted that, when interviews were conducted in the presence of another person, average weekly alcohol consumption was lower, compared to interviews conducted in private. Similar findings were reported by Edwards et al. [264], but only among males. Several studies of adolescent reporting of alcohol and drug use also found that the presence of a parent during a household interview reduces respondent willingness to report such behaviors [127, 265–268]. In contrast, Hoyt and Chaloupka [127] also reported that the presence of friends during an interview increased substance use reporting, and Aquilino et al. [266] reported that the presence of a spouse or significant other had no effect on reports of alcohol and drug use. It is important to recognize, though, some potential confounding, as those most likely to have another person present during an interview are those who are married, and those who have children, and these variables are also commonly associated with less substance use behavior.

The physical context within which interviews take place may also influence social desirability pressures and self-report quality. Much of this evidence comes from comparisons of adolescent survey responses when the surveys are completed at home versus in a school setting. In school settings, parental monitoring is likely to be perceived as less of a concern and confidentiality assurance likely to be more credible. Findings support this supposition, as Brener et al. [132] and others [21, 269–271] have reported that adolescents will underreport substance use during household surveys, relative to school-based surveys. Needle and colleagues [272] and Zanes and Matsoukas [273], though, did not find differences in the reports obtained from students in school- versus home-based settings.

### 4.3. Processing Errors

Once data collection is complete, the construction of a final survey data set requires the implementation of numerous coding and editing rules. The integrity of these rules is particularly critical in substance use surveys, as they typically involve assumptions about the reporting intentions and substance use behaviors of respondents. Fendrich and Johnson [274] have documented important differences in the editing assumptions made across national surveys of substance use in the US that can substantially influence the prevalence estimates generated by each.

Investigators also use a variety of techniques to screen completed substance use questionnaires for inclusion in final data files. Farrell and colleagues [207] examined the effects of excluding respondents (1) who provided a large number of inconsistent answers and (2) who reported use of a fictitious substance. The effects of excluding these responses on prevalence estimates were considered to be minimal, although they cautioned that exclusionary criteria should be used carefully in order to avoid producing nonrepresentative results.

Also, a past report by the US General Accounting Office [6] identified imputation problems in the National Household Survey on Drug Abuse in which the estimated number of past year heroin users in the US ranged from 232,000 to 701,000, as a consequence of whether missing data imputation procedures were or were not used. The same report also indicated that sample weights used to construct subgroup estimates of the total number of illicit drug users were in some instances based on extremely small numbers of individuals in some weighting cells who reported current drug use. In one case from the 1991 NHSDA, a single 79-year-old woman was projected to represent approximately 142,000 persons believed to have used heroin during the previous year. In such instances, a single erroneous data entry could be expected to have dramatic effects on overall survey estimates.

### 4.4. Inferential Errors

Inferential errors can be avoided by insuring that the survey questions being employed and the respondents being sampled are representative of the constructs and populations to which the researcher plans to make inferences. To the degree that either the measures or sample fail to represent their intended objects, inferential errors will be realized. Avoiding inferential errors also entails employing sound research designs and appropriate analytic procedures. Experimental findings are considered the strongest evidence for internal validity, and representative samples provide the strongest evidence for external validity. When research designs deviate from these ideals or measures do not adequately assess the constructs of interest, there is a risk of inferential errors that will limit the generalizability of empirical findings. In substance use research, errors of inference can be of several types. Some are a consequence of erroneously concluding that associations between constructs do not exist, due to poor measures and/or research designs. Others involve falsely concluding that associations do exist between constructs when they in fact do not, also as a consequence of inadequate designs and/or measures. The failure to properly adjust a high quality substance use survey for its stratified sample design, discussed earlier in Section 3.4, is an example of an adjustment error that led to a serious inferential error when investigators erroneously concluded that a large fraction of all alcohol sales in the US were being made to underage minors.

## 5. Discussion

Over several decades, considerable knowledge has been accumulated regarding sources of error in the survey assessment of substance use behaviors. Important gaps remain, however, and continued research will be necessary. Below, I highlight some of the most important questions that I see relevant to each source of survey errors that have been considered in this paper.

Regarding coverage errors, the challenge of constructing representative sample frames for both adolescents and adults continues to increase as electronic communications platforms further diversify. This is a general problem that afflicts all survey research efforts but one that can be particularly problematic for substance use research given the associations between these behaviors and likelihood of being covered by many of the potential sources of sample frames. Identification of supplemental frames that might provide better coverage of heavy substance users and which could be employed, with appropriate weights, as supplements to more traditional sample frames when conducting population surveys should be considered.

When survey estimates are reported, sampling errors, in the form of standard errors or confidence intervals, are commonly included. Although reporting these errors is important to survey transparency, it is important to recognize that sampling errors make strong assumptions that can seldom be met in practice. Most importantly, they assume the absence of all other sources of survey error. Given the unlikeliness of this assumption, merely reporting sampling errors can leave survey consumers with a false sense of the precision of survey estimates, as any sampling errors could be completely overwhelmed by any measurement and/or nonresponse errors, for example, in practice. Understanding how sampling errors in substance use surveys may be influenced by other sources of survey error thus seems to be an important research question to be addressed in the future.

Nonresponse errors seem to be another permanent concern that substance use surveys will need to continually address. Of course, the degree to which nonresponse may bias survey findings will vary from topic to topic and question to question. Given the strong associations detected between substance use and nonresponse patterns, it appears that this error source is also particularly relevant for surveys on this topic. An important issue for additional research is the relative usefulness for substance use surveys of the various nonresponse bias analytic strategies reviewed earlier in this paper. Similarly, research into the relative efficacy of various types of adjustments for nonresponse and other forms of error in substance use surveys would seem to be an important future research topic.

In general, there has been little research into specification errors in substance use surveys. This is an oversight, given general acknowledgment that researchers and potential respondents do not always have a shared understanding of the behaviors being examined. Development of strategies for identifying and investigating potential errors of specification is another research topic in need of attention.

It is my personal opinion that the multiple sources of measurement errors reviewed earlier in this paper pose the greatest threat to the accurate assessment of substance use behaviors. There are several practical questions that remain unresolved, such as the predictive power of social desirability measures, the reasons why experienced interviewers appear to obtain fewer reports of substance use behaviors, and the degree to which adolescents might actually overreport their use of alcohol and/or other drugs. Perhaps even more important, how these widely diverse sets of measurement errors interact with one another is poorly understood and remains largely unexamined. Evaluation of how various sources of measurement errors in substance use surveys interact together to influence survey estimates should be a priority for future research.

In terms of processing errors, surveys concerned with substance use would appear on the surface to be no more vulnerable than other types of survey research. Yet, the complexity of most substance use questionnaires, combined with greater item nonresponse rates in many instances, likely provide greater risks for processing errors that can be linked to complex editing rules and assumptions. A general rule of thumb is that the likelihood of experiencing processing errors is inversely associated with the amount of documentation provided with a survey, as careful documentation is an important indicator of quality research. Continued research into the veracity of data editing decision rules, particularly when handling missing data and/or inconsistent self-reports in substance use surveys, would certainly be welcomed.

As with all other sources of survey related error, inferential errors are not unique to substance use surveys. They are in general a product of poor study design and execution that can seriously limit the value of otherwise commendable efforts. A key to addressing potential inferential errors in research is replication. Study findings take on additional credibility and are accorded stronger inference to the degree that they can be replicated in subsequent investigations. Substance use researchers should seek opportunities to replicate findings from other researchers when conducting their own original studies. And journal editors can provide additional service to science by finding ways to make space available for publishing replication studies that are essential to addressing problems of inferential errors that may otherwise go undetected.

It is important to note that the review presented in this paper was not based on a systematic database search. Rather, it is based on the author's personal familiarity with and experience working with this literature over the past several decades. This should be recognized as a limitation.

Finally, it is strongly recommended that substance use researchers who plan to employ survey research methods recognize and report on their efforts to address each of the potential sources of survey related error discussed in this paper. Developing strategies to systematically and rigorously confront each source of errors and transparently sharing one's successes and failures remains the best approach to minimizing the effects of each when using survey methods to investigate substance use patterns and behaviors.

## Figures and Tables

**Table 1:** Elements of the Lavrakas (2013) total survey error model.

Errors of representation	Errors of measurement
Coverage errors	Specification errors
Sampling errors	Measurement errors
Nonresponse errors	Processing errors
Adjustment errors	Inferential errors
